# Pentacyanoammineferrate-Based Non-Enzymatic Electrochemical Biosensing Platform for Selective Uric Acid Measurement

**DOI:** 10.3390/s21051574

**Published:** 2021-02-24

**Authors:** Won-Yong Jeon, Chang-Jun Lee, Tun Naw Sut, Hyug-Han Kim, Young-Bong Choi

**Affiliations:** 1 School of Chemical Engineering, Biomedical Institute for Convergence, Sungkyunkwan University, Suwon 16419, Korea; powerwy@skku.edu (W.-Y.J.); suttunnaw@skku.edu (T.N.S.); 2 Department of Chemistry, College of Natural Science, Dankook University, Anseo-Dong, Cheonan, Chungnam 31116, Korea; chang5654@naver.com (C.-J.L.); hankim@dankook.ac.kr (H.-H.K.)

**Keywords:** uric acid, pentagonal metal complex, non-enzymatic electrochemical biosensor, nickel ion

## Abstract

The electrochemical-based detection of uric acid (UA) is widely used for diagnostic purposes. However, various interfering species such as ascorbic acid, dopamine, and glucose can affect electrochemical signals, and hence there is an outstanding need to develop improved sensing platforms to detect UA with high selectivity. Herein, we report a pentagonal mediator-based non-enzymatic electrochemical biosensing platform to selectively measure UA in the presence of interfering species. The working electrode was fabricated by electrodepositing polymerized 1-vinylimidazole (PVI), which has an imidazole ligand, onto indium tin oxide (ITO), and then conjugating nickel ions to the PVI-coated ITO electrode. Electrode performance was characterized by cyclic voltammetry (CV) and electrochemical impedance spectroscopy (EIS) measurements and integrated together with pentacyanoammineferrate, which can bind to the amine groups of UA and function as an electron transferring mediator. The experimental results showed a wide linear range of UA concentration-dependent responses and the multi-potential step (MPS) technique facilitated selective detection of UA in the presence of physiologically relevant interfering species. Altogether, these findings support that pentacyanoammineferrate-based non-enzymatic electrodes are suitable biosensing platforms for the selective measurement of UA, and such approaches could potentially be extended to other bioanalytes as well.

## 1. Introduction

Uric acid (UA), also named 2,6,8-trihydroxypurine, is the final product of purine metabolism that is related to various diseases such as gout, hyperuria, obesity, diabetes, hypertension, and kidney and heart problems [1]. UA can be used as a biomarker [2], and determining its concentration can help diagnose the abovementioned diseases. The previous literature has reported that the range of UA concentration is between 1.49 to 4.46 mM in urine for a healthy human [3]. In the past years, various determination techniques have been used to measure UA concentration, such as reversed phase liquid chromatography [4,5,6,7], colorimetric and ratiometric fluorescence [8], and flow injection analysis (FIA) [9]. However, these techniques have drawbacks, as their processes are complex and time-consuming, and it is hard to miniaturize the system. In some of these aspects, the electrochemical measurements are more advantageous since they are easy to use and inexpensive, as well as provide specificity and fast monitoring [10,11,12].

The electrochemical measurements have been mainly used for diagnostic sensors in the medical field [13,14,15,16]. The most representative electrochemical diagnostic sensor is a glucose sensor, which can detect glucose level in the blood [17]. Generally, most glucose sensors in the market are enzyme-based electrochemical sensors [18] and have issues with signal detection due to their thermal and chemical instability, and enzyme degradation over time [19]. In addition, the electrodes in enzyme-based sensors can become unstable upon long-term storage in refrigerated conditions.

For these reasons, many researchers have recently been interested in developing the non-enzymatic electrochemical sensors [20] to increase the performance. Nanostructured non-enzymatic sensors with high surface area that are made of metal, metal oxide, carbon nanotube (CNT), and conductive polymers have been developed [21]. Particularly, the electrochemical non-enzymatic UA sensors help minimize the problem of UA oxidation at the enzyme electrode [22], and the use of conductive materials such as metal nanoalloy, graphite, and conducting polymers for electrodes have been useful [23,24,25,26]. However, the low selectivity arising from oxidation of other interfering species, such as ascorbic acid (AA), dopamine (DA), and glucose, at the electrode still remains an issue.

In this regard, the metal hexacyanoferrates-based electrodes are promising [27]. Nickel (II) hexacyanoferrate-based electrodes, which can transfer electrons between the electrode and the sample, have been applied in cholesterol and UA sensors due to their ability to be modified on the conducting polymer or multi-wall carbon nanotube (MWCNT) surface [27,28,29,30]. Metal hexacyanoferrates can be electrodeposited easily by potentiostat, and the electrodes show redox reversibility and reproducibility [23]. However, there is still no discussion about interferences from other species, so the low selectivity still remains a challenge.

In this study, we developed a metal pentacyanoammineferrate-based non-enzymatic UA sensor with minimized interferences and thus increased selectivity. To fabricate the enzyme-free UA sensor, the pentacyanoammineferrate was conjugated onto the indium tin oxide (ITO) electrode modified with nickel (II) ion-absorbed polyvinyl imidazole (PVI). Our electrode is disposable, and we have characterized its performance by the following electrochemical potentiostat techniques: Cyclic voltammetry (CV), electrochemical impedance spectroscopy (EIS), and multi-potential step (MPS). We found optimized pentacyanoammineferrate composition and system to measure UA. Our findings can be used to support the development of UA biosensor for healthcare applications.

## 2. Materials and Methods

### 2.1. Chemicals and Reagent

Potassium hexacyanoferrate(II) trihydrate (ACS reagent, 98.5~102.0%), potassium hexacyanoferrate(III) (ACS reagent, ≥99.0%), uric acid (UA, ≥99%), nickel chloride(II) (23.0~26.0% Ni basis), dopamine (DA, ≥95%), ascorbic acid (AA, ≥99.0%), urea (ACS reagent, 99.0~100.5%), potassium chloride (KCl, ≥99.0%), sodium chloride (NaCl, ≥99.5%), and glucose (ACS reagent) were purchased from Sigma-Aldrich Co. (Milwaukee, WI, USA). Ammonium disodium pentacyanoammineferrate dehydrate (≥95.0%) was purchased from Fluka. Phosphate buffered saline (PBS, 4.3 mM sodium dihydrogen phosphate (NaH_2_PO_4_), 15.1 mM sodium hydrogenphosphate (Na_2_HPO_4_), and 140 mM NaCl) and all other solutions were prepared using deionized (DI) Milli-Q water (Millipore, Japan). Poly(1-vinylimidazole) (PVI) was synthesized as previously described [31]. Analytical reagents were used without further purification.

### 2.2. Electrochemical Measurements

CHI660B potentiostat (CH Instrument Inc., Austin, TX, USA) was used for all electrochemical experiments in which 0.5 mm Pt wire was a counter, Ag/AgCl (3.0 M KCl, Cypress, Lawrence, KS, USA) a reference, and 6 mm diameter ITO electrode a working electrode. The CV and EIS were used for optimizing the electrodes. A mixed solution of 2 mM potassium hexacyanoferrate (II) trihydrate (in 0.5 M KCl) and 2 mM potassium hexacyanoferrate(III) (in 0.5 M KCl) for EIS measurements at the set frequency range from 10^4^ to 10^−2^ Hz and the amplitude of 5 mV. For the determination of UA, CV and MPS experiments were performed. In the MPS measurements, the initial potential was set at 0 V for 2 s, and subsequent measurements were made every 0.1 V for 5 s from 0.4 to 0.8 V.

### 2.3. Fabrication of Nickel (II) Ions- and PVI-Modified ITO Electrode (Ni-PVI-ITO Electrode)

**Scheme 1:**
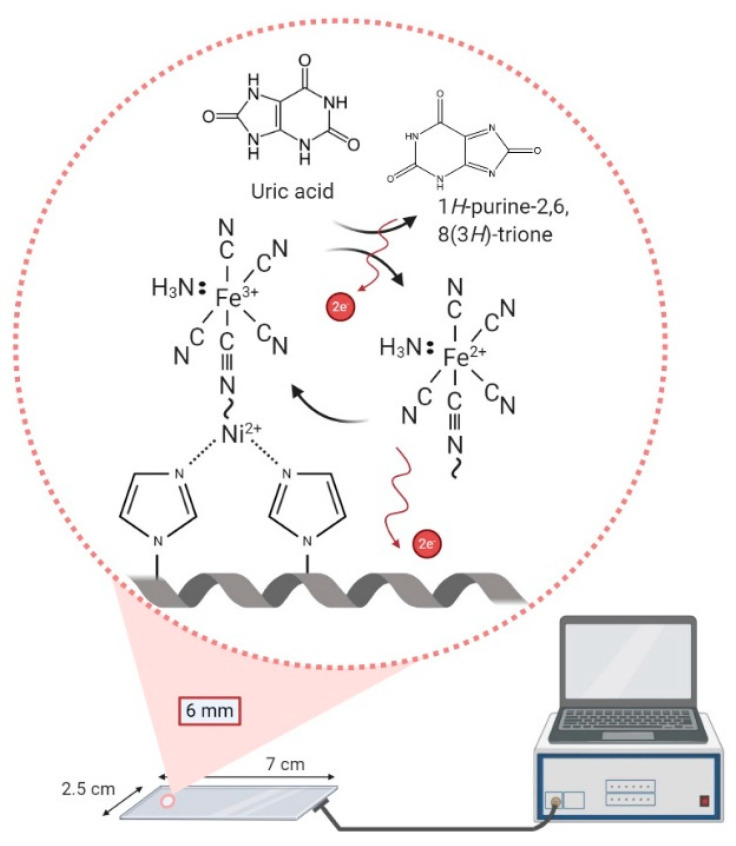
Uric acid electron transfer reaction with pentacyanoammineferrate on Ni-PVI (polymerized 1-vinylimidazole)-ITO (indium tin oxide).

The ITO electrode was immersed in piranha solution (H_2_O_2_:H_2_SO_4_ = 3:1, *v*:*v*) for 5 min and then washed with DI water, followed by 5 min sonication and drying with nitrogen gas. Next, 40 μL PVI (in 1X PBS) was cast onto the cleaned ITO electrode and electrodeposition was carried out with a scan range from −0.4 to 1.0 V at the 100 mV/s scan rate via CV. The PVI-modified ITO electrode was then washed with DI water and the nickel ions were conjugated by casting 40 μL nickel chloride onto it. The Ni-PVI-ITO electrode was then washed with DI water. All electrochemical experiments were carried out on the Ni-PVI-ITO electrodes by using both 20 μL of 0.1 mM ammonium disodium pentacyanoammineferrate dehydrate solution and 20 μL of UA in borate buffer (pH 9.0). The electrochemical reaction of UA with pentacyanoammineferrate on the Ni-PVI-ITO electrode is depicted in Scheme 1.

### 2.4. Optimization of Ni-PVI-ITO Electrode Modification Procedures

To optimize the Ni-PVI-ITO electrode, various PVI concentrations (0.1, 1.0, 5.0, 10.0, and 15.0 mg/mL in 1X PBS), CV cycles (5, 10, 15, and 20), nickel chloride concentrations (0, 1.0, 5.0, 10.0, 15.0, and 20.0 mg/mL in 1X PBS), and nickel chloride reaction times (1, 5, 10, and 20 min) were investigated. All optimization experiments were carried out by the CV technique with both 20 μL of 0.1 mM ammonium disodium pentacyanoammineferrate dehydrate solutions and 20 μL borate buffer (pH 9.0).

### 2.5. Uric Acid Determination

To determine the concentration of UA on the Ni-PVI-ITO electrode, CV experiments were carried out with different solutions containing 20 μL of 0.1 mM ammonium disodium pentacyanoammineferrate dehydrate and 20 μL of 1.0 mM UA in different buffers (acetate, phosphate, or borate buffer). To investigate the effects of interferences, six physiologically relevant interfering species, AA, DA, glucose, urea, KCl, and NaCl were added. CV measurements were carried out at a scan rate of 0.1 V/s and scan range from −0.4 to 1.0 V. The level of UA was then quantified by CV and MPS measurements in solution containing 0.1 mM AA, 0.1 mM DA, 5.5 mM glucose, 50.0 mM urea, 100.0 mM KCl, and 269.0 mM NaCl. The interfering species and their concentrations were chosen based on previous reports [24,32,33,34,35].

## 3. Results and Discussion

### 3.1. Optimization of ITO Modification with Ni and PVI; Ni-PVI-ITO

**Figure 1:**
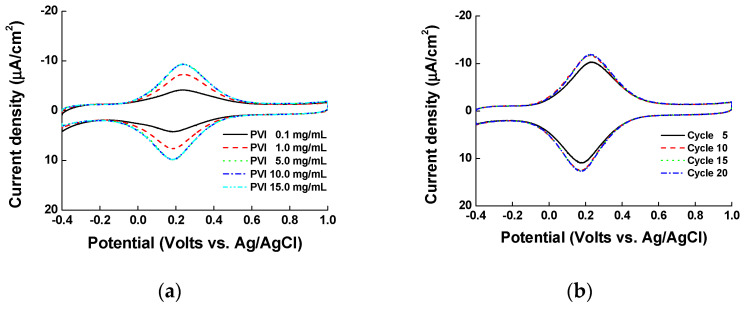
Cyclic voltammograms of electrode optimization in terms of (**a**) PVI concentration (0.1, 1, 5, 10, and 15 mg/mL were tested) and (**b**) electrodeposition cycles (5, 10, 15, and 20 cycles were performed).

**Figure 2:**
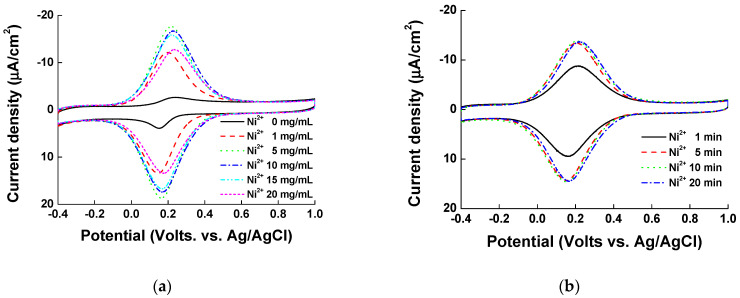
Cyclic voltammograms of electrode optimization in terms of (**a**) nickel ion concentration (0, 1, 5, 10, 15, and 20 mg/mL were tested) and (**b**) reaction time (1, 5, 10, and 20 min were tested).

Before casting the nickel ions, the PVI polymer was electrodeposited onto ITO electrode by CV. Firstly, we tested different concentrations of PVI polymer ranging from 0.1 to 15.0 mg/mL to find the optimal PVI concentration for electropolymerization. The PVI was dissolved in 1X PBS (pH 7.0), and 10 CV cycles were performed. As shown in Figure 1a, the current density reached saturation at 5 mg/mL PVI. Secondly, we checked different electrodeposition cycles of PVI (5, 10, 15, and 20 cycles) at 5 mg/mL PVI (the minimum concentration of PVI for maximum current density determined in the first set of experiments). Before measurements, PVI was incubated in 5 mg/mL nickel chloride for 5 min. As shown in Figure 1b, a total of 10 cycles was sufficient to achieve the maximum current density. Finally, we casted different concentrations of nickel chloride solution (0.0 to 20.0 mg/mL), and allowed different reaction times from 1 to 20 min. In the second and final sets of experiments, we checked the CV results by using 40 μL of pentacyanoammineferrate (in 1X PBS). If there was no nickel ion on the PVI-ITO electrode, the minimal pentacyanoammineferrate peak was detected, as there was minimal binding of pentacyanoammineferrate to the electrode (Figure 2a). In fact, the maximum current density was obtained at 5 mg/mL nickel ion and interestingly, the current density decreased at nickel ion concentration above 5 mg/mL. We reason that if there is a high amount of nickel ions on the PVI-ITO electrode, the binding of pentacyanoammineferrate will be weak due to the low Ni-imidazole conjugation. As shown Figure 2b, a 5 min reaction time in nickel chloride solution was enough to obtain the maximum current density. Taken together, the parameters for the optimized electrode condition are as follows: PVI concentration of 5 mg/mL, 10 cycles of electrodeposition, 5 mg/mL of nickel, and 5 min of nickel reaction time.

### 3.2. Electrochecmial Characterization of Ni-PVI-ITO Electorde

**Figure 3:**
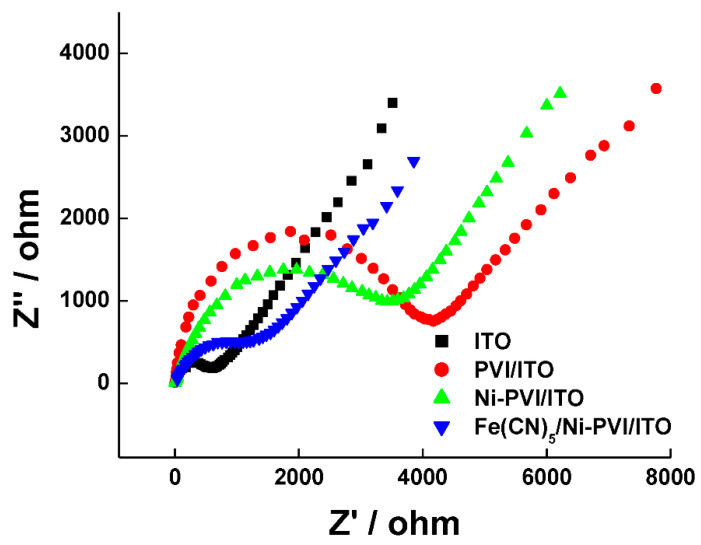
Nyquist plot for bare ITO (black squares), PVI-ITO (red circles), Ni-PVI-ITO (green triangles), and Fe(CN)_5_-Ni-PVI-ITO (blue reverse triangles) electrodes.

The interface properties of the modified electrodes were confirmed electrochemically. In a Nyquist plot of EIS data, the semicircular part at higher frequencies corresponds to an electron-transfer-limited process, and the linear part at lower frequencies corresponds to a diffusion-controlled process. The semicircle diameter indicates the electron-transfer resistance (*R*_et_) [Ret] [36]. The impedance spectra of the bare ITO, PVI-ITO, Ni-PVI-ITO, and Fe(CN)_5_-Ni-PVI-ITO electrodes were investigated in 0.5 M KCl solution containing 2.0 mM K_3_[Fe(CN)_6_]/K_4_[Fe(CN)_6_]. As shown in Figure 3, the *R_et_* of PVI-ITO (red circle) dramatically increased over that of bare ITO (black square), from 687 to 4092 Ω. On the other hand, the *R_et_* of Ni-PVI-ITO (green triangle) was 3950 Ω, which is smaller than that of PVI-ITO due to the effect of nickel metal ion. The Fe(CN)_5_-Ni-PVI-ITO (blue inverted triangle) had an even smaller *R_et_* of 1666 Ω due to the conductive redox mediator of ferrate. This result suggests that the PVI polymer blocks the electron transfer of the electrode, while Fe(CN)_5_ and nickel ion help increase the conductivity of the electrode.

### 3.3. Interference Species Testing

**Figure 4:**
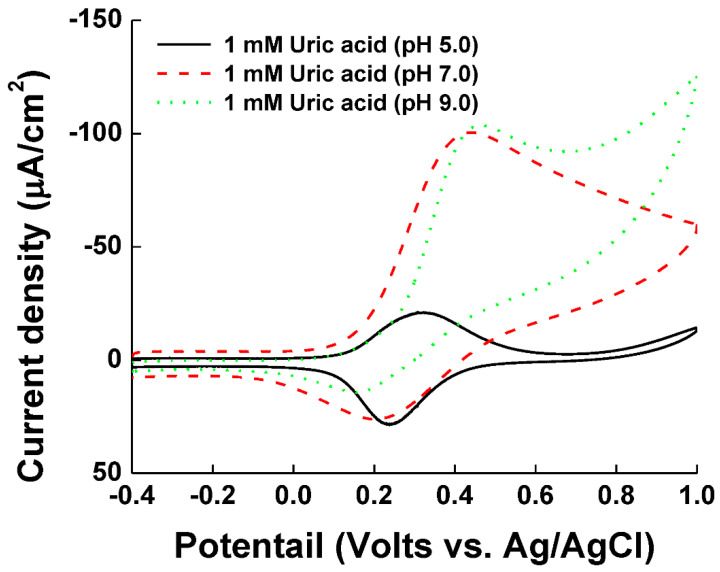
Cyclic voltammograms of measuring 1 mM uric acid with various buffers such as acetate (pH 5.0), phosphate (pH 7.0), and borate (pH 9.0).

The optimized Ni-PVI-ITO electrode was used for the determination of UA (1.0 mM) with pentacyanoammineferrate. As shown Figure 4, UA showed low catalytic current in acetate buffer with pH 5.0 (black line), but significantly higher catalytic currents in phosphate with pH 7.0 (red dash) and borate buffer with pH 9.0 (blue dot). UA has a pKa of 5.35 and hence, it is hard to dissociate in pH 5.0. It has been previously reported that the dissociation of UA in pH 5.0 is less than 10%, while the complete dissociation occurs at pH above 6.5 [37,38]. Therefore, the anion formed from UA dissociation can easily approach the nearby Ni-PVI-ITO surface and then transfer the electrons with pentacyanoammineferrate.

**Figure 5:**
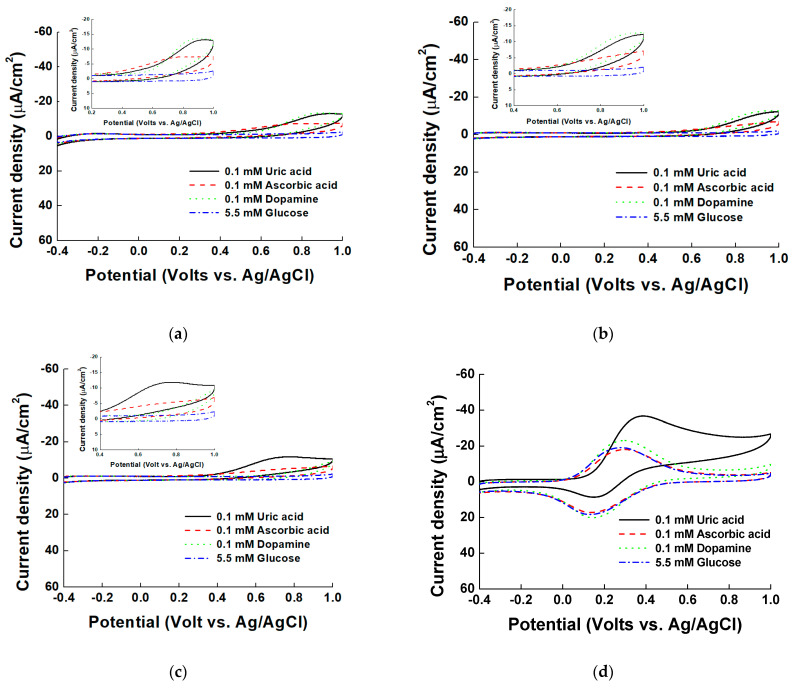
Cyclic voltammograms of 0.1 mM uric acid (UA), 0.1 mM AA, 0.1 mM DA, and 5.5 mM glucose in borate buffer (pH 9.0) with (**a**) ITO, (**b**) PVI-ITO, (**c**) normal Ni-PVI-ITO, and (**d**) pentacyanoammineferrate based Ni-PVI-ITO. The insets in (**a**–**c**) show the magnified results.

In addition, the four amines of UA likely coordinate with pentacyanoammineferrate and get oxidized on the Ni-PVI-ITO electrode. When the interference species (0.1 mM AA, 0.1 mM DA, and 5.5 mM glucose) were added, the measurement signals were insignificant for all the tested electrodes (ITO, PVI-ITO, and Ni-PVI-ITO) without pentacyanoammineferrate (Figure 5a–c) in borate buffer (pH 9.0). Moreover, the oxidation voltage was too high (around 0.6 to 0.8V). Figure 5d shows the cyclic voltammograms of measurements for UA, AA, DA, and glucose with pentacyanoammineferrate. The catalytic current of UA was observed at the 0.4 V potential. However, all interferences showed appreciable catalytic currents as well so UA measurement in our system (performed in PBS-based solution; see also Figure S1) was not selective. Note that UA, DA, and AA are all deprotonated in PBS-based solution (pH 7.0) and therefore can interact with positively charged nickel (II) ions. However, in strong base solution of borate buffer, the negative charge of pentacyanoammineferrate prevents the interaction of the interfering species with electrodes, and only UA can interact with the electrode because it has four amine groups. This confirms the role of the basic condition of borate buffer alongside amine groups in enabling UA to be oxidized selectively. We also measured protein denaturants and electrolytes by adding 50.0 mM urea, 269.0 mM sodium ion, and 100.0 mM potassium ion in borate buffer, but could not observe any oxidation peak (Figure S2). This further confirms the suitability of borate buffer for the selective UA measurement.

**Figure 6:**
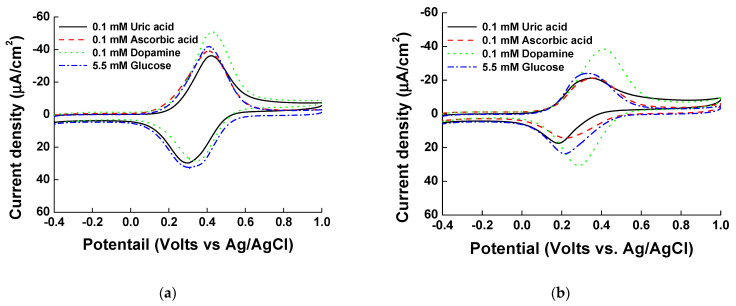
Cyclic voltammograms of 0.1 mM UA, 0.1 mM AA, 0.1 mM DA, and 5.5 mM glucose with hexacyanoferrate Fe(CN)_6_-based Ni-PVI-ITO in (**a**) pH 7.0 and (**b**) pH 9.0.

Besides the buffer condition, it is noteworthy that the pentacyanoammineferrate has the unstable pentagonal structure and thus can play a role in UA oxidation. To investigate this role, we performed the same experiments with hexacyanoferrate, which has the stable hexagonal structure and found that UA measurement was not selective in both phosphate (Figure 6a) and borate (Figure 6b) buffers. This demonstrates the pentagonal structure, not the hexagonal structure, of ferrate is key to UA selectivity, while the basic condition of borate buffer is the second important factor, but not mutually exclusive. Therefore, our system with pentacyanoammineferrate in borate buffer can measure UA selectively in the presence of interference species under physiological conditions.

### 3.4. Uric Acid Measurement on Ni-PVI-ITO Electrode

**Figure 7:**
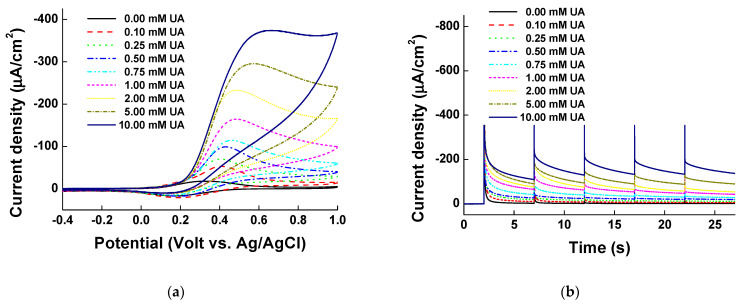

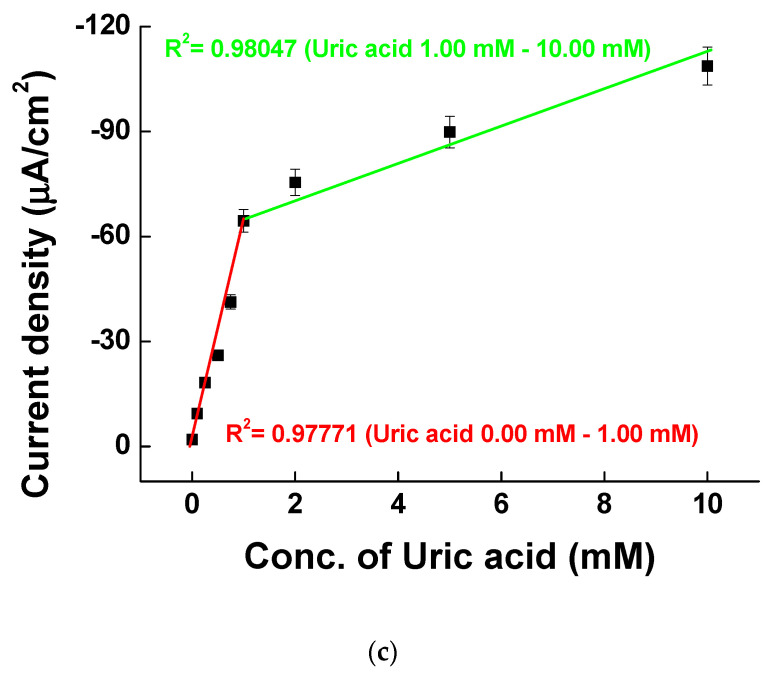
(**a**) Cyclic voltammograms and (**b**) multi-potential step (MPS) results of measuring uric acid at concentrations of 0.00, 0.10, 0.25, 0.50, 0.75, 1.00 mM, 2.00, 5.00, and 10.00 mM in borate buffer (pH 9.0). (**c**) Calibration curve of uric acid concentration from 0.0 to 10.00 mM at 0.4 V of MPS in (**b**).

**Scheme 2:**
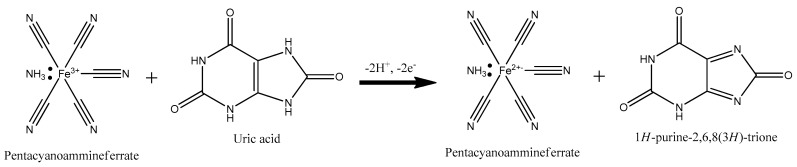
Electrochemical mechanism of uric acid with pentacyanoammineferrate.

Different UA concentrations of 0.00, 0.10, 0.25, 0.50, 0.75, 1.00, 2.00, 5.00, and 10.00 mM were tested by CV and MPS for the Ni-PVI-ITO electrode with pentacyanoammineferrate in borate buffer (pH 9.0). The cyclic voltammograms of UA (Figure 7a) show the catalytic currents over 0.4 V, with linear dependence on the UA concentration. The catalytic currents are a result of electron transfer between UA and pentacyanoammineferrate. Scheme 2 outlines the proposed mechanism of electron transfer process between pentacyanoammineferrate and UA.

The electrons from UA oxidation are transferred to pentacyanoammineferrate to reduce Fe^3+^ to Fe^2+^. The electrons from reduced pentacyanoammineferrate are then moved to the electrode for making catalytic currents. Figure 7b shows the MPS results of various UA concentrations at 0.4, 0.5, 0.6, 0.7, and 0.8 V. The first 2 s is for quiet time and afterwards, the voltage was changed every 5 s from 0.4 to 0.8 V. Figure 7c shows the calibration curve at 0.4 V of MPS measurements vs. UA concentration. The sensitivity was higher at low UA concentration range between 0.00 and 1.00 mM (with a slope of −57.99 μA/mM and a correlation coefficient R^2^ of 0.97771) than at high UA concentration range between 1.00 and 10.00 mM (with a slope of −4.88 μA/mM and a correlation coefficient R^2^ of 0.94200). The limit of detection (LOD) was 0.05 mM (*n =* 5) and thus, our system has good performance for quantitative UA measurements for diagnosis.

### 3.5. Uric Acid Measurement on Ni-PVI-ITO Electrode in the Presence of Interferences

**Figure 8:**
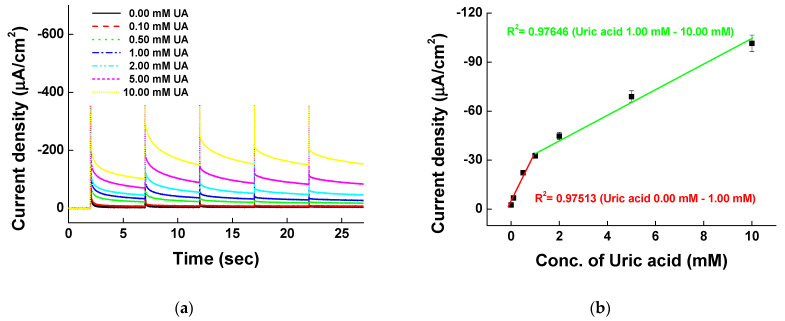
(**a**) MPS graph and (**b**) calibration curve of uric acid (0.00 to 10.00 mM) in solution containing all interfering species of 0.1 mM AA, 0.1mM DA, 5.5 mM glucose, 50.0 mM urea, 100.0 mM KCl, and 269.0 mM NaCl in borate buffer (pH 9.0).

The reliability of the fabricated Ni-PVI-ITO electrode in the presence of physiologically relevant interfering species—0.1 mM AA, 0.1 mM DA, 5.5 mM glucose, 50.0 mM urea, 100.0 mM KCl, and 269.0 mM NaCl—was analyzed. Figure 8a shows the MPS results of measurements with 0.00, 0.10, 0.50, 1.00, 2.00, 5.00, and 10.00 mM UA in solution of interfering species. UA oxidized well, as indicated by the currents at 0.4 V increasing with UA concentration. The oxidation of UA with pentacyanoammineferrate on the Ni-PVI-ITO electrode was selective, and the calibration curve showed different sensitivities between low and high UA concentrations (Figure 8b) with a trend similar to that of UA measurement without interfering species (cf. Figure 7c). The slope of the curve was higher at 0.00–1.00 mM UA (−30.29 μA/mM with R^2^ = 0.97513) than at 1.00–10.00 mM (−7.93 μA/mM, R^2^ = 0.97646), indicating that the sensitivity is greater at low UA concentrations. Overall, the Ni-PVI-ITO electrode can be used for selective UA sensing in the presence of the interfering species.

### 3.6. Uric Acid Measurement in Urine Samples

**Figure 9:**
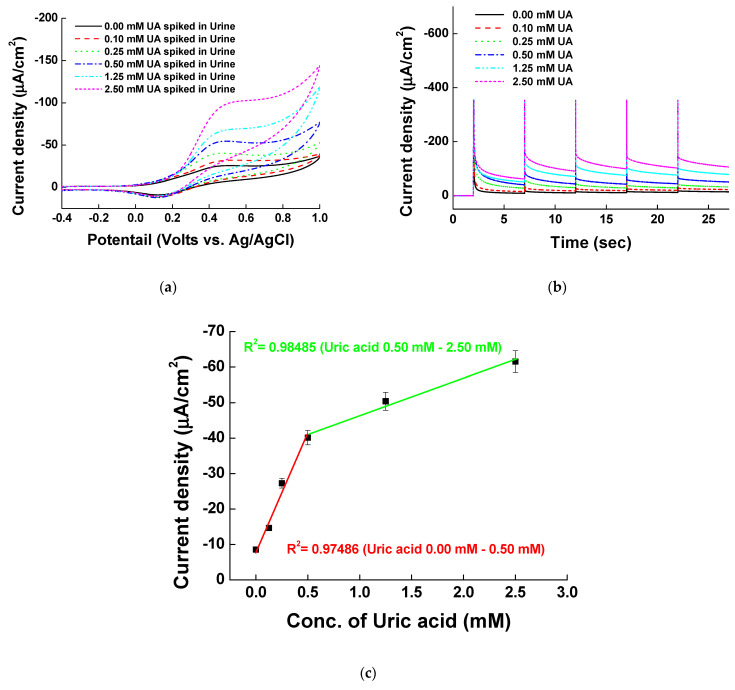
(**a**) Cyclic voltammograms and (**b**) MPS results of measuring uric acid at concentrations of 0.000, 0.125, 0.250, 0.500, 1.250, and 2.500 mM in urine sample. (**c**) Calibration curve of uric acid concentration from 0.000 to 2.500 mM at 0.4 V of MPS in (**b**).

Electrochemical signals were measured by spiking UA to healthy adult urine samples in the prepared Ni-PVI-ITO electrode. Measurements were carried out by adding 20 μL of 0.2 mM pentacyanoammineferrate, 10 μL of unpurified urine sample, and 10 μL of UA (0.00 to 2.50 mM) in borate buffer (pH 9.0). Urine samples were provided by two healthy adults, and experiments were conducted with five sets of electrodes (*n* = 5) to confirm reproducibility. The results of CV and MPS measurements of UA-spiked urine samples are shown in Figure 9a,b, respectively. There was a direct correlation between the current density signals and UA concentration in urine, which further demonstrates the selectivity of our UA measurement system. Figure 9c shows the calibration curve of MPS signals vs. spiked UA concentration. As expected, the sensitivity (slope) was higher at UA concentration below 1.00 mM. Table 1 compares the performance of our non-enzymatic electrochemical UA sensor to that of others reported in the literature. The advantages of our sensing system include the ability to detect a wide UA concentration range that is medically relevant, the ease with which it can be applied in commercialized biosensors, and the novelty of using pentacyanoammineferrate as the electron-transferring platform at the electrode. Additionally, intra-assay was performed and the analysis indicates reproducibility and accuracy of our UA sensing platform (Table 2). Overall, our system that is newly fabricated Ni-PVI-ITO electrode with pentacyanoammineferrate showed excellent performance for determining the concentration of UA.

## 4. Conclusions

UA is the primary end product of purine metabolism, and quantitative analysis of UA in body fluids is an important indicator associated with the prevention of diseases. Moreover, the development of an electrochemical UA sensor, which provides easy sensing and reliable quantitative results, is very important. In this study, we developed a non-enzymatic UA sensor. We first optimized the electrode fabrication conditions regarding PVI concentration, nickel ion concentration, electrodeposition cycles of PVI, and reaction time of nickel, and found them to be 5 mg/mL, 5 mg/mL, 10 cycles, and 5 min, respectively. Moreover, we analyzed the measurement of UA in the presence of various interfering species (AA, DA, glucose, urea, sodium ion, and potassium ion) with pentacyanoammineferrate in borate buffer. We found that the selectivity for UA is due to the pentagonal structure of pentacyanoammineferrate as well as the basic condition of borate buffer, allowing the oxidation of UA at the electrode. The key role of the pentagonal structure in conferring selectivity was evaluated against the hexacyanoferrate. The oxidative catalyst currents produced by UA were linearly proportional to UA concentration, showing good performance of our system as an electrochemical sensor for the quantification of UA from 0.00 to 10.00 mM with LOD of 0.05 mM (*n* = 5). In addition, the MPS data showed that the maximum electrochemical currents of UA were detected at 0.4 V in the presence of interfering substances. The measurement signals obtained with urine samples spiked with different concentrations of UA were also linearly proportional to spiked UA concentration, which indicates the practical utility of our platform for real biological samples. Taken together, we have demonstrated that our system can be used for the development of enzyme- and label-free electrochemical diagnostic sensors to quantify UA, while its reusability remains to be improved.

## Figures and Tables

**Table 1:** Comparison of our non-enzymatic electrochemical UA sensor with other reports.

Electrode	Technique	Electrolyte and pH	Potential Applied (V)	Linear Range (mM)	LOD (μM)	Ref.
PAMTA/PG	DPV	PB 7	0.92	0.005 to 1.2	2.74	[39]
β-CD/MWNT-CILE	LSV	AB 5	0.5	0.0006 to 0.4 and 0.4 to 1.0	0.3	[40]
WS_2_/GCE	DPV	PBS 7.2	0.24	0.005 to 1.0	1.2	[41]
*p* (ABSA)/GCE	SWV	0.1 M NaOH	−0.132	0.02 to 0.2	5.9	[42]
Ni-PVI-ITO	MPS	BB 9.0	0.4	0.1 to 10.0	50	Our report

**Table 2:** Determination of UA using the fabricated electrode and validation using MPS from the borate buffer and spiked urine sample (*n* = 3).

Assay	Sample	Nominal Concentration (mM)	Calculated Concentration (mM)	RSD (%)	Accuracy (%)
Intra	Borate buffer	0.100	0.1243	0.0004	124.27 ± 0.0005
		1.000	1.0491	0.0687	104.91 ± 0.068
		10.000	11.6699	4.879	116.69 ± 4.88
	Spiking in Urine	0.125	0.113	0.031	90.289 ± 0.12
		1.250	1.17	0.44	93.593 ± 0.45
		2.500	2.168	0.3	86.717 ± 0.31

## Data Availability

Not applicable.

## Supplementary Materials

The following are available online at https://www.mdpi.com/1424-8220/21/5/1574/s1, Figure S1: Cyclic voltammograms of measuring uric acid and various interferences in pH 7.0 buffer, Figure S2: Cyclic voltammograms of interfering species such as 0.1 mM UA, 0.1 mM AA, 0.1 mM DA, 5.5 mM glucose, 50.0 mM urea, 100.0 mM KCl, and 269.0 mM NaCl in borate buffer (pH 9.0) in pentacyanoammineferrate based Ni-PVI-ITO.
