# Supplementary material for: Pentacyanoammineferrate-Based Non-Enzymatic Electrochemical Biosensing Platform for Selective Uric Acid Measurement
Source: Sensors (Basel). 2021 Feb 24;21(5):1574. doi: 10.3390/s21051574 (PMC7956465; doi:10.3390/s21051574)
Supplement: Supplementary file 1 [file sensors-21-01574-s001.pdf]

sensors

MDPI

Supplementary data

Pentacyanoammineferrate-Based Non-Enzymatic Electrochemical Biosensing Platform for Selective Uric Acid Measurement

Won-Yong Jeon [1], Chang-Jun Lee [2], Tun Naw Sut [1], Hyug-Han Kim [2] and Young-Bong Choi [2,*]

[1] School of Chemical Engineering, Biomedical Institute for Convergence, Sungkyunkwan University, Suwon, 16419, Korea; powerwy@skku.edu (W.-Y.J.); suttunnaw@skku.edu (T.N.S.)
[2] Department of Chemistry, College of Natural Science, Dankook University, Anseo-Dong, Cheonan, Chungnam 31116, Korea; chang5654@naver.com (C.-J.L.); hankim@dankook.ac.kr (H.-H.K.)
* Correspondence: chem0404@dankook.ac.kr; Tel.:+82-41-550-3437; Fax: +82-41-559-7860

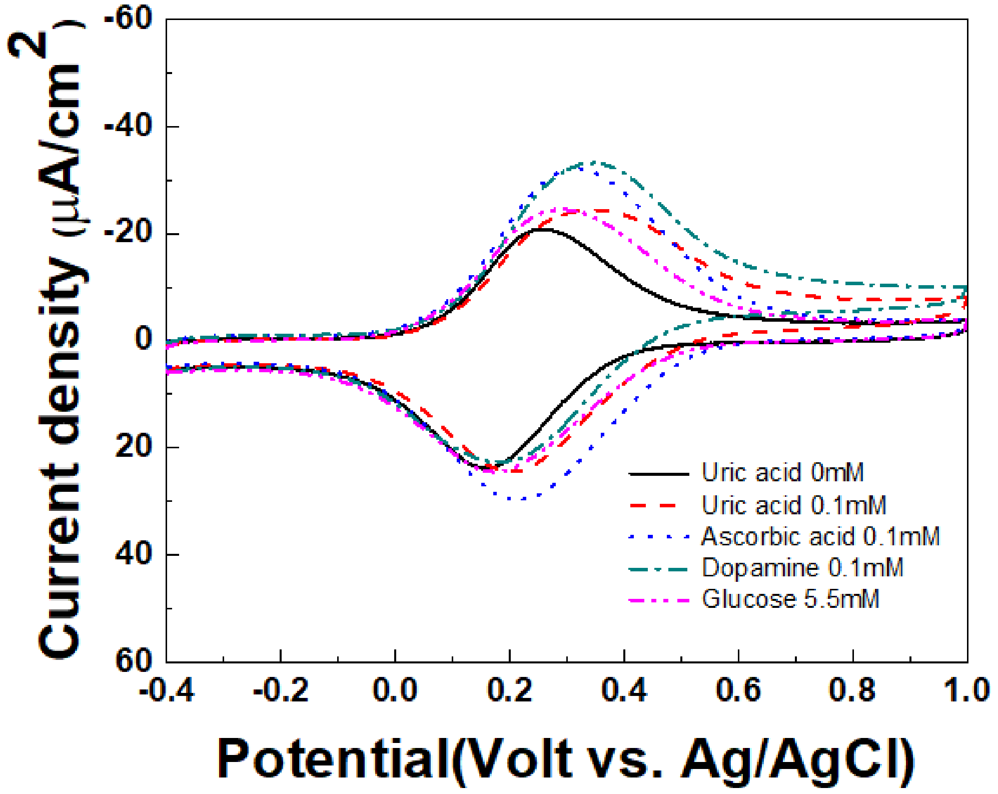

Figure S1. Cyclic voltammograms of measuring uric acid and various interferences in pH 7.0 buffer.

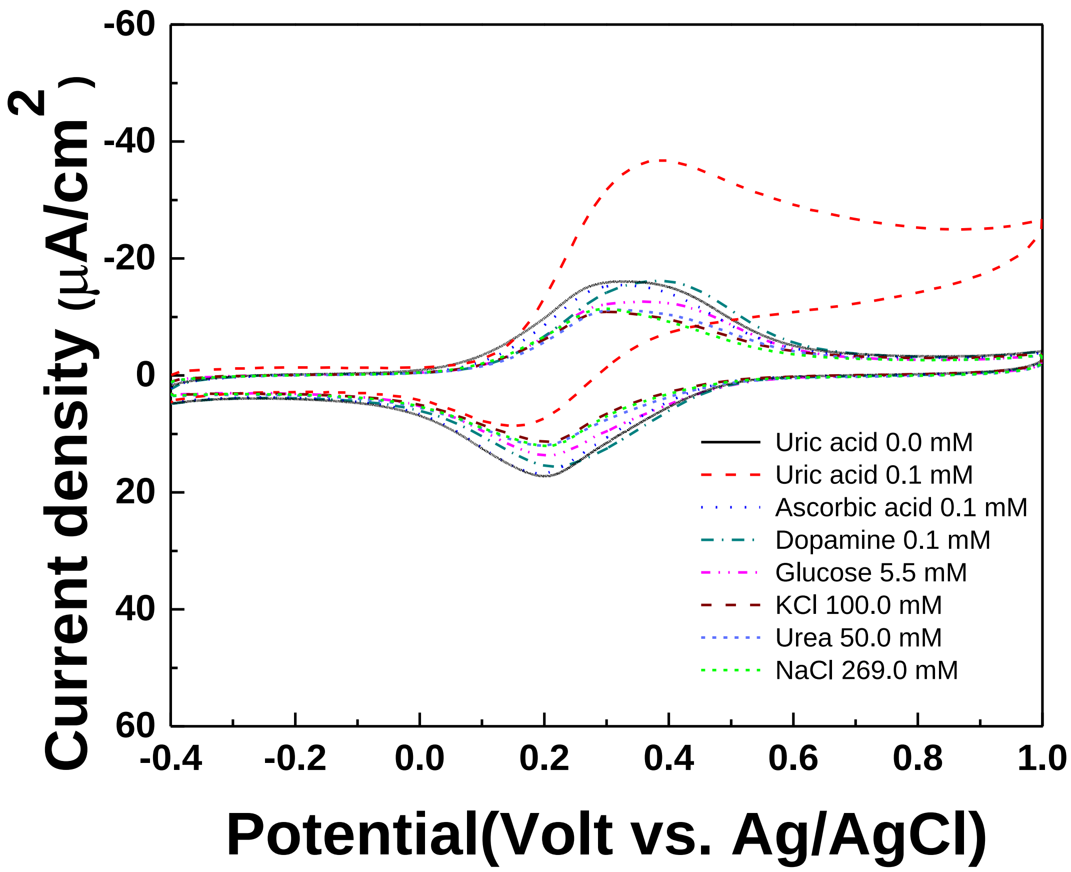

Figure S2. Cyclic voltammograms of 0.0 mM & 0.1 mM UA, 0.1 mM AA, 0.1 mM DA, 5.5 mM glucose, 50.0 mM urea, 100.0 mM KCl, and 269.0 mM NaCl in borate buffer (pH 9.0*)* with pentacyanoammineferrate-based Ni-PVI-ITO.
